# Complexity in clinical diagnoses of acute exacerbation of chronic obstructive pulmonary disease

**DOI:** 10.1186/s12890-023-02587-1

**Published:** 2023-08-14

**Authors:** Alexandre J. Pratt, Andrew Purssell, Tinghua Zhang, Vanessa P. J. Luks, Xavier Bauza, Sunita Mulpuru, Miranda Kirby, Shawn D. Aaron, Juthaporn Cowan

**Affiliations:** 1 Department of Medicine, University of Ottawa, Ottawa, ON Canada; 2 Division of Infectious Diseases, Department of Medicine, University of Ottawa, Ottawa, ON Canada; 3 Clinical Epidemiology Program, The Ottawa Hospital Research Institute, Ottawa, ON Canada; 4 Division of Respirology, Department of Medicine, University of Ottawa, Ottawa, ON Canada; 5 Department of Physics, Toronto Metropolitan University, Ottawa, ON Canada

**Keywords:** Acute exacerbation, Chronic obstructive pulmonary disease, Diagnosis

## Abstract

**Background:**

Acute exacerbation of chronic obstructive pulmonary disease (AECOPD) is a clinical syndrome with various causes. It is not uncommon that COPD patients presenting with dyspnea have multiple causes for their symptoms including AECOPD, pneumonia, or congestive heart failure occurring concurrently.

**Methods:**

To identify clinical, radiographic, and laboratory characteristics that might help distinguish AECOPD from another dominant disease in patients with a history of COPD, we conducted a retrospective cohort study of hospitalized patients with admitting diagnosis of AECOPD who were screened for a prospective randomized controlled trial from Sep 2016 to Mar 2018. Clinical characteristics, course in hospital, and final diagnosis at discharge were reviewed and adjudicated by two authors. The final diagnosis of each patient was determined based on the synthesis of all presenting signs and symptoms, imaging, and laboratory results. We adhered to AECOPD diagnosis definitions based on the GOLD guidelines. Univariate and multivariate analyses were performed to identify any associated features of AECOPD with and without other acute processes contributing to dyspnea.

**Results:**

Three hundred fifteen hospitalized patients with admitting diagnosis of AECOPD were included. Mean age was 72.5 (SD 10.6) years. Two thirds (65.4%) had spirometry defined COPD. The most common presenting symptom was dyspnea (96.5%), followed by cough (67.9%), and increased sputum (57.5%). One hundred and eighty (57.1%) had a final diagnosis of AECOPD alone whereas 87 (27.6%) had AECOPD with other conditions and 48 (15.2%) did not have AECOPD after adjudication. Increased sputum purulence (OR 3.35, 95%CI 1.68–6.69) and elevated venous pCO2 (OR 1.04, 95%CI 1.01 – 1.07) were associated with a diagnosis of AECOPD but these were not associated with AECOPD alone without concomitant conditions. Radiographic evidence of pleural effusion (OR 0.26, 95%CI 0.12 – 0.58) was negatively associated with AECOPD with or without other conditions while radiographic evidence of pulmonary edema (OR 0.31; 95%CI 0.11 – 0.91) and lobar pneumonia (OR 0.13, 95%CI 0.07 – 0.25) suggested against the diagnosis of AECOPD alone.

**Conclusion:**

The study highlighted the complexity and difficulty of AECOPD diagnosis. A more specific clinical tool to diagnose AECOPD is needed.

**Supplementary Information:**

The online version contains supplementary material available at 10.1186/s12890-023-02587-1.

## Introduction

Acute exacerbations of chronic obstructive pulmonary disease (AECOPD) are associated with increased morbidity, mortality, and healthcare cost [1–3]. The 2022 GOLD guidelines define AECOPD as an acute worsening of respiratory symptoms that results in additional therapy [4]. However, it is increasingly recognized that AECOPD is heterogenous. Exacerbations have a range of underlying etiologies and clinicopathological processes manifesting with similar syndromic phenotypes, each of which could have different prognosis and response to therapy [5]. A diagnosis of AECOPD relies on subjective symptoms, and non-specific clinical features, which can be confounded by several associated comorbidities that are highly prevalent in the COPD population [6]. It is not uncommon that COPD patients presenting with dyspnea receive a diagnosis of AECOPD, pneumonia, and congestive heart failure all in one setting [7, 8]. This ambiguity leads to a broad, complex, and potentially unnecessary treatment regimen.

We aimed to identify clinical, radiographic, and laboratory characteristics that might help distinguish AECOPD from symptoms secondary to another dominant disease in patients with a history of COPD.

## Methods

### Study population/setting

We conducted a retrospective cohort study of patients identified from a prospective pilot randomized controlled trial (IPRAC) that aimed to determine feasibility of monthly intravenous immunoglobulin infusions for one year to prevent recurrent AECOPD in hospitalized patients with AECOPD from Sep 2016 to Mar 2018 [9]. The IPRAC study received institutional approval from the Ottawa Health Science Network Research Ethics Board (Protocol 20150925-01H, 20160077-01H, and 2017005-01H), which allowed for retrospective analysis of collected data from patients screened for eligibility. For the current study, we included only hospitalized patients age > 40 years old with an admitting diagnosis of AECOPD. Patients with history of active malignancy, clear alternative admitting diagnosis such as failure to cope, documented refusal to participate in research, death prior to March 2018, admission only to the Emergency Department but not to an Inpatient service, and absence of smoking history were excluded.

### Data collection

The electronic medical records of patients included in our retrospective cohort were divided equally to be reviewed and extracted in a systematic manner by AJP, AP, and SA. AP and JC independently reviewed the extracted data and determined the final diagnosis based on discharge diagnosis and documentation which included a synthesis of all presenting signs and symptoms, imaging, and laboratory results available over the admission. Discrepancy of the final diagnosis was then discussed between AP and JC to agree on a final diagnosis. We categorized final diagnosis into AECOPD alone or AECOPD with other conditions, or no AECOPD. Our terminology is an elaboration of that published in the *2022 Gold Reports—Global Initiative for Chronic Obstructive Lung Disease (GOLD).* AECOPD alone was considered if there was no evidence of other acute or comorbid conditions that could have contributed to the patient’s presentation of acute respiratory symptoms. Of note, we included instances where a respiratory viral process such as influenza was present in the AECOPD alone group because the GOLD guidelines define respiratory viral infections as a common trigger of AECOPD and the symptoms experienced by the patient are generally due to the AECOPD and not the triggering viral infection [10]. AECOPD with other conditions was considered when dyspnea and wheezes were accompanied by other identifiable etiologies such as pulmonary edema, pulmonary embolism, or evidence of pneumonia. When there was clear evidence of bacterial pneumonia such as the presence of fever, leukocytosis, and lobar consolidation, we classified this as pneumonia, not AECOPD with pneumonia. Concomitant pneumonia was considered when the clinical presentations met AECOPD criteria but the patient also had fever, and/or leukocytosis, and/or new radiographic infiltrations. Patients who did not have “acute” worsening symptoms of at least 2 of the following symptoms – dyspnea, sputum production, or cough – were classified as no AECOPD.

### Data analysis

We described the distribution of baseline demographic and clinical characteristics of patients using numbers (proportions) and means [standard deviation (SD)], as appropriate. Univariable and multivariable logistic regression models were fit to investigate relationship between i) AECOPD with and without other conditions as the dependent variable, and potential risk factors as independent variables, ii) AECOPD alone as the dependent variable, and potential risk factors as independent variables. Five independent variables (subjective increase in sputum purulence, venous pCO2, radiographic evidence of pleural effusion, pulmonary edema, and lobar pneumonia) in multivariable analysis were selected based on univariate analysis results and clinical importance agreed among study investigators. All statistical analyses were conducted using SAS for windows version 9.4(SAS Institute, Cary, NC).

## Results

### Study patients

**Fig. 1:**
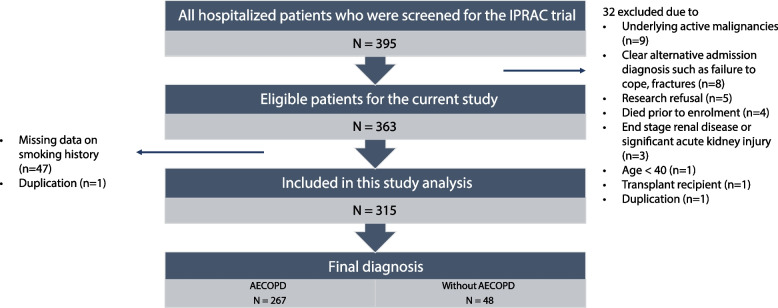
Flow chart of included study patients

There were 395 hospitalized patients with an admitting diagnosis was AECOPD who were screened for the IPRAC trial. Of these, 32 were excluded since they were ineligible for the IPRAC study (9 underlying active malignancies, 8 clear alternative admission diagnosis such as failure to cope or fractures, 5 research refusal, 4 died prior to enrolment, 3 end stage renal disease or significant acute kidney injury, 1 age < 40, 1 transplant recipient, and 1 duplication). The remaining 363 patients underwent electronic chart review of the hospitalization, of whom 1 was a duplicate, and 47 did not have documented information on smoking history and were excluded. As a result, there were 315 patients available for the study analysis. (Fig. 1.)

The inter-observer agreement for the final diagnosis was 87.7% after the initial adjudication and increased to 100% after the final adjudication. Included patients were classified based on the final diagnosis of AECOPD only (*n* = 180), AECOPD with other conditions (*n* = 87), and no AECOPD (*n* = 48). Other conditions that were associated with AECOPD in 87 patients were pneumonia (77/87; 88.5%), congestive heart failure (11/87; 12.6%), and pulmonary embolism (2/87; 2.3%). There were 3 patients who had both pneumonia and congestive heart failure. For the 48 patients who did not have AECOPD as the final diagnosis, 22 (45.8%) had pneumonia, 7 (14.6%) had congestive heart failure with or without atrial fibrillation. Other diagnoses included worsening pulmonary fibrosis, severe stable COPD without evidence of an acute exacerbation, lung cancer, upper airway disease, pulmonary hypertension, anxiety, and pneumococcal sepsis. Mean age was 72.5 (SD 10.6) years. Two thirds (65.4%) had spirometry defined COPD while approximately a quarter had a history of congestive heart failure (25.7%) and/or coronary artery disease (29.5%). The most common presenting symptom was dyspnea (96.5%), followed by cough (67.9%), increased sputum (57.5%), increased purulence of sputum (44.8%), and chest tightness (11.7%). Fever was present in 25.4% of cases. O2 supplementation was given to 43.2% of our patient cohort during triage in the Emergency Department, and 62.5% had wheezes on examination. Bronchodilators, systemic corticosteroids and antibiotics were given to most patients at time of presentation, 94.6%, 92.1%, and 86.0%, respectively. Clinical, laboratory, and radiographic parameters at the time of hospital admission of the entire cohort, and by final diagnosis are summarized (Table 1 and Additional file 1). There was no missing data of the laboratory parameters reported in Table 1.

**Table 1:** Clinical, laboratory, and radiographic data of included patients by group of adjudicated diagnosis at discharge

	**Feature**	**Entire Cohort** *n* = 315	**No AECOPD** *n* = 48	**AECOPD present**		
				Total *n* = 267	AECOPD alone *n* = 180	AECOPD with other conditions *n* = 87
Demographics	Age, mean (SD)	72.5 (10.6)	75.0 (13.1)	72.1 (10.0)	71.5 (9.3)	73.3 (11.3)
	Females, n (%)	189 (60.0)	31 (63.5)	158 (59.2)	115 (63.9)	43 (49.4)
	Smoking history in pack years, mean (SD)	42.5 (28.4)	38.4 (36.7)	43.2 (26.7)	45.3 (26.7)	39.0 (26.5)
	Spirometrically defined COPD, n (%)	206 (65.4)	26 (54.2)	180 (67.4)	130 (72.2)	50 (57.5)
	Asthma, n (%)	24 (7.6)	3 (6.25)	21 (7.9)	17 (9.4)	4 (4.6)
	ILD, n (%)	4 (1.3)	2 (4.16)	2 (0.7)	1 (0.6)	1 (1.2)
	Oxygen supplementation at baseline, n (%)	81 (25.7)	10 (20.8)	71 (26.6)	51 (28.3)	20 (23.0)
	O2 in L	0.73 (1.5)	0.9 (1.7)	0.7 (1.5)	0.76 (1.4)	0.7 (1.6)
	History of CHF, n (%)	81 (25.7)	19 (39.6)	62 (23.2)	37 (20.6)	25 (28.7)
	History of CAD, n (%)	93 (29.5)	15 (31.3)	78 (29.2)	49 (27.2)	29 (33.3)
Symptoms at presentation in ED	Increase in dyspnea, n (%)	304 (96.5)	43 (89.6)	261 (97.8)	177 (98.3)	84 (96.6)
	Increase in sputum, n (%)	181 (57.5)	17 (35.4)	164 (61.4)	110 (61.1)	54 (62.1)
	Increased purulence of sputum, n (%)	141 (44.8)	13 (27.1)	128 (47.9)	81 (45.0)	47 (54.0)
	Increased Cough, n (%)	214 (67.9)	31 (64.6)	193 (72.3)	126 (70.0)	67 (77.0)
	Chest tightness, n (%)	37 (11.7)	4 (8.3)	33 (12.4)	25 (13.9)	8 (9.2)
Physical exam findings	SPO2% on triage, mean (SD)	90.3 (7.55)	90.3 (6.31)	90.3 (7.76)	90.6 (8.14)	89.6 (6.9)
	O2 in triage, n (%)	136 (43.2)	9 (18.75)	127 (47.6)	91 (50.6)	36 (41.4)
	Fever, n (%)	80 (25.4)	16 (33.3)	64 (24.0)	40 (22.2)	24 (27.6)
	Wheeze on exam, n (%)	197 (62.5)	25 (52.1)	172 (64.4)	113 (62.8)	59 (67.8)
Treatment initiated in ED	Bronchodilator given, n (%)	298 (94.6)	37 (77.1)	261 (97.8)	176 (97.8)	85 (97.7)
	Steroid given, n (%)	290 (92.1)	32 (66.7)	258 (96.6)	175 (97.2)	83 (95.4)
	Antibiotics given, n (%)	271 (86.0)	40 (83.3)	231 (86.5)	148 (82.2)	83 (95.4)
CXR findings	Pneumothorax, n (%)	1 (0.3)	–-	1 (0.4)	–-	1 (1.2)
	Pleural effusion, n (%)	53 (16.8)	17 (35.4)	36 (13.5)	18 (10.0)	18 (20.7)
	Lobar pneumonia, n (%)	68 (21.6)	8 (16.7)	60 (22.5)	19 (10.6)	41 (47.1)
	Atypical pneumonia, n (%)	12 (3.8)	4 (8.3)	8 (3.0)	4 (2.2)	4 (4.6)
	Pulmonary edema, n (%)	28 (8.9)	11 (23.0)	17 (6.4)	8 (4.4)	9 (10.3)
	Consider pneumonia, n (%)	80 (25.4)	15 (31.3)	65 (24.3)	27 (15.0)	38 (43.7)
Lab values	WBC, mean (SD)	11.9 (8.0)	13.0 (5.4)	11.7 (8.4)	11.3 (9.3)	12.6 (6.0)
	Blood neutrophils, mean (SD)	8.76 (4.3)	10.2 (4.9)	8.5 (4.1)	7.9 (3.6)	9.6 (4.8)
	Eosinophils (10^9/L), mean (SD)	0.19 (0.3)	0.12 (0.2)	0.2 (0.3)	0.2 (0.3)	0.1 (0.2)
	Venous pCO2 (mmHg), mean (SD)	57.1 (16.8)	51.1 (14.0)	58.2 (17.0)	59.5 (18.3)	55.4 (13.8)

*ILD* Interstitial lung disease, *CHF* Chronic heart failure, *HFrEF* Heart failure with reduced ejection fraction, *HFpEF* Heart failure with preserved ejection fraction, *CAD* Coronary artery disease, *CXR* Chest x-ray

### Clinical characteristics associated with a diagnosis of AECOPD with or without other conditions vs. those without AECOPD

Univariate analysis of all extracted variables revealed that increased dyspnea (OR 5.05; 95% CI 1.48 – 17.24), increased sputum (OR 2.91; 95%CI 1.53 – 5.52), increased purulence of sputum (OR 2.48; 95%CI 1.26 – 4.90), and venous pCO2 level (OR 1.03; 95% CI 1.01 – 1.06) were positively associated with AECOPD, while history of CHF (OR 0.46; 95%CI 0.24 – 0.88), radiographic evidence of pleural effusion (OR 0.28; 95%CI 0.14 – 0.57), and pulmonary edema (OR 0.23; 95%CI 0.10 – 0.53) were negatively associated with AECOPD. The results of multivariable analysis which included the five prespecified covariates are shown in Table 2. Subjective increase in sputum purulence (OR 3.35; 95%CI 1.68 – 6.69), and venous pCO2 (OR 1.04; 95% CI 1.01 – 1.07) were predictors for AECOPD while radiographic evidence of pleural effusion suggested against AECOPD (OR 0.26; 95%CI 0.12 – 0.56).

**Table 2:** Association between clinical features and AECOPD

Feature	Univariate analysis OR, 95%CI	Multivariate analysis^a^ OR, 95%CI
Subjective increase in sputum purulence	2.48 (1.26 – 4.90)	3.35 (1.68 – 6.69)
Venous pCO2	1.03 (1.01 – 1.06)	1.04 (1.01 – 1.07)
CXR—pleural effusion	0.28 (0.14 – 0.57)	0.26 (0.12 – 0.58)
CXR—pulmonary edema	0.23 (0.01 – 0.53)	0.47 (0.18 – 1.24)
CXR—lobar pneumonia	1.45 (0.64 – 3.26)	1.83 (0.76 – 4.46)

^a^adjusted for age, subjective increase in sputum purulence, venous pCO2, CXR findings of pleural effusion, and pulmonary edema

### Clinical characteristics associated with AECOPD alone, vs patients with AECOPD and other conditions

Univariate analysis of all extracted variables revealed that female sex (OR 1.81; 95%CI 1.08 – 3.04) and spirometrically defined COPD (OR 1.92; 95%CI 1.13 – 3.29) were positively associated with having the diagnosis of AECOPD alone, while blood eosinophilia (OR 0.91; 95%CI 0.85 – 0.97), radiographic evidence of pleural effusion (OR 0.43; 95%CI 0.21 – 0.87), and lobar pneumonia on CXR (OR 0.13; 95%CI 0.07 – 0.25) were negatively associated with this outcome. In the multivariable analysis, radiographic evidence of pulmonary edema (OR 0.31; 95%CI 0.11 – 0.91) and lobar (OR 0.15; 95%CI 0.08 – 0.29) was negatively associated with the diagnosis of AECOPD alone (Table 3).

**Table 3:** Association between clinical features and AECOPD alone

Feature	Univariate analysis OR, 95%CI	Multivariate analysis OR, 95%CI
Subjective increase in sputum purulence	0.70 (0.42 – 1.16)	0.86 (0.47 – 1.56)
Venous pCO2	2.66 (0.74 – 9.62)	1.01 (0.99 – 1.03)
CXR—pleural effusion	0.43 (0.21 – 0.87)	0.53 (0.23 – 1.18)
CXR—pulmonary edema	0.40 (0.15 – 1.08)	0.31 (0.11 – 0.91)
CXR—lobar pneumonia	0.13 (0.07 – 0.25)	0.13 (0.07 – 0.25)

## Discussion

Our study highlights the challenge of diagnosing AECOPD. The data indicates a limited utility of clinical and/or laboratory findings to distinguish classical AECOPD from AECOPD associated with other acute processes/illnesses. Among the 3 symptoms listed in the clinical criteria of AECOPD diagnosis, all were associated with AECOPD in univariate analysis. However, none of the clinical symptoms analyzed could differentiate AECOPD alone from AECOPD with other acute process. Not surprisingly, our study found that elevated venous pCO2 was associated with a diagnosis of AECOPD while radiographic findings of pleural effusion suggested against AECOPD, and radiographic evidence of pulmonary edema or pneumonia suggest against AECOPD only diagnosis. This is consistent with existing literature [11, 12]. Evidence of pneumonia on imaging strongly suggests that the AECOPD is likely related to lower respiratory tract infection [11].

Interestingly, approximately 12% of patients in our study did not have AECOPD by our adjudication criteria but were labeled as such on admission. Unfortunately, there is limited literature available on the incidence of misdiagnosis of exacerbation, but this does highlight the challenge of making a diagnosis of AECOPD. One reason the diagnosis of AECOPD can be so challenging may be related to frequency of misdiagnosis of COPD in the general population. In Ontario, only one third of patients in the general population labeled with COPD have received spirometry to confirm the diagnosis [13]. Current literature suggests a third to over half of patients identified as having COPD in the primary care setting may be misdiagnosed [14, 15]. Although we might expect this to be decreased in the setting of hospitalized patients given their increased contact with the healthcare system and likely more severe disease, literature suggests that a third of these patients are also misdiagnosed with COPD [16]. Even among frequent exacerbators with diagnosed asthma and COPD, a quarter were found to be misdiagnosed [17]. As for our study, despite a third of our study patients not having spirometry-defined COPD, many received a diagnosis of AECOPD and were treated as such. As a result, the prevalence of misdiagnosis in hospitalized patients and the general population make it even more challenging to identify characteristics unique to AECOPD to help distinguish it from other underlying etiologies.

Our determination of whether a patient’s presentation of AECOPD was accompanied by another condition was potentially limited by subjectivity since a reference standard is lacking in the field. The manifestations of AECOPD are protean, non-specific, and could represent a variety of non-AECOPD conditions. The GOLD report recommends that differential diagnoses are excluded before making a diagnosis of AECOPD [4], which is often impractical especially considering that AECOPD and other etiologies can present concurrently. A superior definition would align a specific criteria-based diagnosis of AECOPD with prognosis and response to therapy, yet no such standard exists at this time.

Increased sputum purulence was shown to be associated with the presence of bacteria in the lower respiratory tract [18]. A recent systematic review and meta-analysis also showed a moderate level of evidence that sputum purulence is associated with a significantly higher probability of potentially pathogenic bacteria [19]. Although we did not examine if increased sputum purulence was associated with presence of bacteria in the lower respiratory tract, we found that increased sputum purulence might be associated with AECOPD with co-existing other condition, not with AECOPD only, which is in keeping with the existing literature. A prospective non-randomised interventional pilot study applying a sputum purulence-guided strategy of antibiotic treatment reported no difference in treatment failure between those with purulent sputum treated with antibiotics versus those with non-purulent sputum not treated with antibiotics [20]. The 2020 GOLD highlights the importance of sputum purulence in the decision to prescribe antibiotics for AECOPD. In our cohort, antibiotics were often deployed as part of treatment (86%). Their use was the highest in patients deemed to have AECOPD with other conditions present (95.4%), which includes patients diagnosed with concurrent bacterial pneumonia. Yet, antibiotic use remained high in those patients deemed to be presenting with AECOPD only (82.2%), despite only a fraction of the individuals presenting with evidence of bacterial infection such as purulent sputum (45%). Moreover, sputum purulence in this study was reported by patients, not assessed by health-care providers, which can be less sensitive and specific for the presence of bacteria [21]. Hence, antibiotic overuse maybe more than we expected. Our finding further highlights the complexity of AECOPD diagnosis.

The study’s major limitation was the small sample size which limited our ability to fit many variables in the multivariate analysis. Other limitations include the subjective adjudication of definition based on clinical presentation and retrospective nature of the study which did not allow for assessment of detailed time course of clinical symptoms and laboratory parameters. Additionally, some laboratory parameters such as C-reactive protein, procalcitonin, BNP, or D-dimer which could help discern AECOPD from AECOPD with other acute processes/illnesses were not included because these tests were not consistently measured in all study patients.

This study highlighted the difficulty of AECOPD diagnosis. We identified increased sputum purulence and venous pCO2 to be predictors for AECOPD, but pleural effusion on chest radiographs suggested against AECOPD. A more accurate and specific clinical tool to diagnose AECOPD with different phenotypes is needed.

### Supplementary Information

**Additional file 1**. File 1 Study Raw Data. Study raw data used for the analysis was organized into 4 spreadsheets. The first sheet contains data for 267 patients diagnosed with AECOPD as their final diagnosis. The second sheet contains data for 180 patients diagnosed solely with AECOPD. The third sheet contains data for 87 patients diagnosed with both AECOPD and other conditions. Lastly, the fourth sheet contains data for 48 patients who do not have a final diagnosis of AECOPD.

## Data Availability

The dataset supporting the conclusions of this article is available within the paper and Supplementary File 1.
